# The Venereal

**Published:** 1840-01-01

**Authors:** 


### THE VENEREAL.

The Modern Treatment of Syphilitic Diseases, both Primary and Secondary: comprising an Account of the New Remedies, with numerous Formulæ for their Preparation, and Mode of Administration. By *Langston Parker*, Member of the Royal College of Surgeons, Fellow of the Royal Medical and Chirurgical Society of London, and Lecturer on Anatomy and Physiology in the Birmingham Royal School of Medicine and Surgery, &c. &c. London, Churchill, 12mo. pp. 158.

One would suppose that all the world was poxed, so many works on it does the press teem with, so many lectures on it are the weekly journals infected with. We wish we could say that all the works and all the lectures display much acquaintance with it. We suspect that it is on—

> " The value of a thing
> Is just what it will bring,"

principle, that syphilis is much in vogue at present. Be that as it may, we must notice these productions as they are produced, and Mr. Parker's happens to lie just now before us.

Mr. Parker informs us in his Preface:—

" The modern researches on Syphilis, its complications and consequences, and the improved modes of practice which have been the result of such investigations in this country, and on the continent, have, as yet, been made known to the public only in detached portions, and that chiefly, if not altogether, through the medium of the periodical medical literature of the day.

Much attention to the subject has impressed me with a conviction, that a work containing the *result* of modern experience, would not be unacceptable to the profession; whilst, to the junior part of it, such a publication might be exceedingly useful.

Usefulness, and not originality, has been the great object I have consulted in composing the present work. It contains little, and I believe no theoretical matter, except perhaps the account of Mons. Ricord's researches 'On Inoculation,' in reference to Syphilis. I originally intended, and to the best of my ability I have carried out my intention, that it should contain only the result of generally received modern experience on the treatment of Syphilitic Diseases.

I have adopted no party in the question; as will be perceived by a perusal of the work, not agreeing exclusively with the mercurialists, on the one hand, or condemning the remedy *in toto* on the other, in accordance with the principles of the physiologic school, or the partisans of the simple treatment. I have endeavoured to hold out to the confidence of the reader those plans of treatment, and those only which are calculated to cure his patient the most speedily and with the greatest safety."

The work is, in point of fact, a compilation, and that mainly from French writers. We must therefore be excused from offering any thing like an analysis of it. We may content ourselves with stating that it consists of sixteen sections, and that they appropriate to themselves the following matters. Section 1, of the simple treatment of syphilitic diseases—of the simple constitutional treatment of syphilis—of the simple, local, or surgical treatment of syphilis—of the mercurial treatment of syphilitic diseases; section 2, of particular preparations of mercury, and their mode of employment—mercurial fumigations, the proto-chloride of mercury, the bichloride of mercury, the proto-ioduret of mercury, the cyanuret of mercury, the deuto-phosphate of mercury—section 3, on inoculation, as applied to the diagnosis, and treatment of syphilitic diseases—section 4, of the first class of syphilitic diseases—gonorrhœa, its varieties and consequences—of balanitis, gonorrhœa, symptoms, sympathies, varieties, consequences and terminations—treatment—of specific remedies—copaiba, particular forms for its exhibition—cubebs, particular forms for its exhibition—of mercury—of injections; section 5, of diseases which succeed to gonorrhœa in the male; section 6, of gonorrhœa in the female—of the second class of primary syphilitic diseases—ulcers, their varieties and consequences; section 7, of chancres of the urethra; section 8, of phagedænic primary venereal sores—the local treatment of phagedæna—of mercury—of the simple treatment of phagedæna—of bubo; section 9, of constitutional or secondary syphilis; section 10, of syphilitic diseases of the skin—of mercury—of the use of iodine and its preparations—sudorifics—the muriate of gold; section 11, of the particular forms of syphilitic diseases of the skin—syphilitic exanthemata, syphilitic squamæ, syphilitic vesiculæ, syphilitic pustulæ, syphilitic papulæ; section 12, of the syphilitic tubercula; section 13, of constitutional syphilitic ulcers—of vegetations; section 14, of the syphilitic testicle—section 15, of pains in the bones; section 16, of periostitis—of ostitis—of the operation for the relief of phimosis.

We shall pick out some formulæ and some facts, both mostly continental, and endeavour to consult the utile rather than the mirabile in our selection.

### 1. *Particular Preparations of Mercury.*

*The Proto-Ioduret of Mercury.*—MM. Cullerier, Biett, and several others, employ the proto-ioduret of mercury in preference to the bichloride, as more certain in its effects and less liable to decomposition. This preparation may be employed in primary syphilitic sores in strumous habits, but is chiefly resorted to in chronic affections and in those varieties of disease which have been described by authors as constitutional syphilis.

M. CULLERIER'S FORM FOR THE EMPLOYMENT OF THE PRO-IODURET OF MERCURY.

℞. Hydrargyri proto-ioduret, gr. xij.

Ext. v. Pulv. Opii, gr. vj

Gummi Guaiaci, ʒ j. M. ft. Pil. xxiv.

Capiat 1 nocte maneque.

POMMADE OF VAL DE GRACE.

℞. Hydrargyri proto-ioduret, ℈ j.

Adipis, ℥ j. M.

Employed in friction upon tumours and indolent buboes after all acute inflammatory symptoms have disappeared.

Mr. Parker goes on to inform us:—

It is principally, says M. Cullerier, in secondary syphilis that the proto-ioduret of mercury is administered with success. Its effects are principally evident in secondary ulceration of the mucous membrane, cutaneous tubercles, exostoses, and chronic affections of the joints, where the other preparations of mercury have had little effect.

The deuto-ioduret of mercury may be employed in friction or in pills, in the same manner as the proto-ioduret: it is stronger and more stimulating, and consequently should be administered in smaller doses. Both these remedies may be advantageously employed in all cases of secondary syphilis, with more certainty and less risk than the deuto-chloride; also in indolent buboes, in obstinate ulcers with hardened base and edges. In all instances they must be guarded by opium, and the patient must be submitted to a strict dietetic and antiphlogistic regimen during their employ. The dose of the deuto-ioduret from $\frac{1}{16}$ of a grain to a grain.

We would observe that recommendations to employ these mercurial preparations in what are called "secondary symptoms" must be carefully scrutinised and cautiously acted on. Four-fifths of "inveterate secondary symptoms" are not secondary symptoms at all. They are cachectic symptoms mostly due to mercury, and aggravated by its further use. And the advice to keep patients on antiphlogistic regimen during the exhibition of medicines of this class is to be received cum grano salis. As a general rule low living and depletory treatment do not answer during a course of mercury. Salivation is more likely to occur, the health is more liable to suffer, and cachectic symptoms more apt to be developed or increased. Certainly it is not in the *treatment* of disease, be it what it may, that we would take Continental practitioners as guides.

*The Cyanuret of Mercury.*—The following account and formulæ are given us by Mr. Parker.

The cyanuret of mercury is now frequently employed in preference to the bichloride, and for the following reasons. It is more soluble and not so liable to decomposition, acts more quickly, and does not occasion those pains in the stomach and bowels that so frequently accompany the prolonged administration of the bichloride. According to the researches of M. Parent, the cyanuret of mercury is not decomposed by either acids or alkalis, nor by decoctions containing azotised principles or gallic acid.

The cyanuret of mercury may be administered internally in pills, or in solution, and used externally in form of pommade or ointment. M. Cullerier employs the cyanuret in primary syphilis. Externally it is an extremely useful application to various forms of herpes, particularly that form termed by Alibert, "herpes squamosus," the violent itching and irritation of which it allays. It may be employed externally also as a dressing to indolent syphilitic ulcers, and scirrhous tubercles, or as a gargle in ulcerations of the throat. The dose of the cyanuret is from $\frac{1}{16}$ of a grain to a grain.

GARGLE OF THE CYANURET OF MERCURY.

℞. Hydrargyri Cyanuret. gr. x.
Infus. Lini. Comp., ℔ j. M.

PILLS OF THE CYANURET OF MERCURY.

℞. Hydrargyri Cyanuret., gr. viij.
Pulv. Opii, gr. xvj.
Ext. Guaiaci, ʒij. M. ft. Pil. lxiv. Capt. 1 ter die.

OINTMENT OF THE CYANURET OF MERCURY.

℞. Hydrargyri Cyanuret, gr. xij.
Adipis, ℥j. M. ft. Unguentum.

SOLUTION OF THE CYANURET OF MERCURY.

℞. Hydrarg. Cyanuret. gr. vj. ad gr. x.
Aquæ, ℔ j. M.

Half an ounce for a dose, administered in a mucilaginous vehicle or with the addition of sugar in form of syrup.

M. PARENT'S CYANURETTED PILLS.

℞. Hydrargyri Cyanuret. gr. xxiv.

Ammoniæ Muriatis, ʒiij.

Guaici Gummi, ℥iij.

Ext. Aconiti, ʒiij.

Ol. Anisi, ♏. xxiv. M. Mucilagini, q. s. ft. Pil. 400.

One or two twice or three times a day, the dose gradually increased. Each pill contains about $\frac{1}{16}$ of a grain of the cyanuret. These pills are a substitute for the bichloride of mercury in many forms of secondary syphilis.

ANOTHER FORM OF M. PARENT.

℞. Hydrarg. Cyanuret., gr. vj.
Opii, gr. xij.
Micæ panis, q. s. ft. Pil. xcvj.

Each pill contains $\frac{1}{16}$ of a grain of the cyanuret adapted to forms of primary syphilis. The dose of one or two pills twice a day in the commencement, and gradually increased.

*Deuto-Phosphate of Mercury.*—The deuto-phosphate of mercury is employed in Italy, more paticularly at Naples, in the treatment of venereal affections in preference to the other salts of this metal. It may be given internally in pills or in solution in the same doses as the bichloride. Employed by friction upon indolent buboes or exostoses, it is said to be preferable to all other forms of the remedy. It is of use also as a dressing to indolent chancres.

OINTMENT OF THE DEUTO-PHOSPHATE OF MERCURY.

℞. Hydrargyri deuto-phosphatis, gr. viij.
Adipis, ℥j. M. ft. Unguent.

In cases of indolent bubo, a few grains are rubbed daily upon the tumour, the frictions also are to be made upon the groin of the opposite side. Our experience in the use of this remedy is drawn chiefly from the practice of Fiore and the Neapolitan surgeons, it is scarcely used in France, although occasionally employed in Germany.

We shall here introduce some of the remedies employed for secondary symptoms.

*Iodine and its Preparations.*—"These remedies are employed largely in the treatment of the secondary and tertiary symptoms of constitutional syphilis. Iodine of itself is a powerful antisyphilitic, but, unless in a state of combination with mercury, is inadmissible in the treatment of the simple primary forms of disease. It may be employed in the cutaneous diseases of constitutional syphilis with great advantage, and, though a remedy not so active as mercury, is particularly indicated in constitutional syphilis, where the primary forms of disease have been treated by full courses of the former medicine. Cullerier uses the following form for its administration.

℞. Iodinii, gr. j.

Potassæ Iodid. gr. ij. ad iv.

Aquæ, ℥j. M.

This may be put into a pint or quart of any vehicle; as the decoction of sarsaparilla, &c. and given at intervals during the day. The dose of the iodine may be increased to two grains in the day, and that of the iodide of potass to six, or ten. The preparations of iodine are chiefly of use in constitutional syphilis, in scrofulous or delicate patients, and in glandular enlargements of syphilitic character, which have resisted the action of mercurials.

M. Ricord employs the iodide of potassium chiefly in those forms of constitutional syphilis which he has termed tertiary. These are tubercles of the skin and mucous membranes, which, in the venereal pathology of this surgeon, form the link connecting the secondary with the tertiary forms of disease, nocturnal pains, periostosis, ostitis, caries, and tumours of the bones: in all these forms of disease, the iodide of potassium is considered by Ricord as the remedy 'par excellence.' He does not rely upon it so much in the secondary affections of the skin, unless used in combination with the iodurets of mercury. The dose of the iodide of potassium, in the commencement, should be ten grains in the day, dissolved in an ounce of distilled water, and administered at intervals in any convenient vehicle; every two or three days the dose may be increased, observing its effects: Ricord increases it ten grains every three days; he has carried it as far as one hundred and forty grains in the day without any ill effect. The iodide of potassium accelerates the pulse, and occasions a slight heat in the stomach; generally, however, if the stomach be free from disease or irritation, it materially improves the digestive powers. If given in an overdose, the heat in the stomach amounts to pain, and may be followed by inflammation; it occasions also when thus employed, pricking or irritation of the skin, followed by a pustular eruption; sometimes the head is affected, and the quantity of urine enormously increased. Ricord mentions a case in which this took place to the extent of forty or fifty pints in the day, it was not found, on analysis, to contain sugar. We shall return to the special indication for the employment of this remedy when speaking of the particular forms of constitutional syphilis." 129.

Certainly, so far as our experience goes, iodine and its preparations are not worthy of any confidence in primary syphilis; and are not deserving of much in the true secondary symptoms. But in the mixed symptoms of cachexia they frequently act almost mavellously. In the rupial or ecthymatous ulcerations of the skin, in the diseases of the bones that sometimes *may* result from syphilis, and often *do* result from mercury, the iodide of potassium is highly beneficial. Yet it seems inconsistent and uncertain in its operation; for, in one case, it will remove the symptoms rapidly and completely, and in another, apparently similar, it will be of only temporary benefit. Nay, in some cases, we have thought that it was in the long run, mischievous, relieving the patient for the time, but rather sapping his constitution eventually.

Of the enormous doses prescribed by M. Ricord we can only say that we should not like to venture on their use. We have never seen anything to prove the utility of excessive doses, and we are sure that they are not unaccompanied with hazard. If ten or fifteen grains a day do no good, more will probably do no good neither, and may, perhaps, do harm. Even moderate doses give rise at times to irritation of the gastro-intestinal mucous membrane.

*The Muriate of Gold.*—"This remedy is particularly useful in the vegetating forms of the syphilides. It is prescribed in the form of ointment; from six to sixteen grains of the salt to an ounce of lard. Biett prefers it to every other remedy, as a local application to syphilitic vegetations. Internally it may be exhibited under the same forms, and in the same doses as the bichloride of mercury, its internal use is however uncertain." 132.

We have tried the muriate of gold internally in several cases, but we cannot say that we ever found it of service.

On the whole, from what we have seen, we believe that, with the exception of the iodide of potassium, these new preparations will disappoint practitioners. The great secret of treating syphilis well consists not in the number of tools, but in understanding the handling of a few good ones.

*Inoculation of Syphilitic Diseases.*—There cannot be a question of the value of inoculation, did circumstances allow of its unexceptionable employment. Inoculation would go far towards settling many litigated questions. M. Ricord has lately experimented very *liberally* with inoculation, and Mr. Parker presents, in a very abridged form, his conclusions. We shall insert the principal here.

" M. Ricord establishes, in the first place, that a chancre, wherever it may be seated, is produced by a specific matter which is secreted by a chancre only, which matter produces a similar disease whenever placed in circumstances favorable to contagion.

This specific matter is only secreted from the surface of a chancre during its first stage, that is, during the period of ulceration, or when the sore is indolent or stationary. At these periods only does a chancre secrete a specific matter capable of producing a similar disease by inoculation. When the sore begins to heal and a process of reparation has commenced, it is merely a simple ulcer, does not furnish a specific secretion, and is not capable of propagation by inoculation.*

If matter be taken from a chancre during the period of ulceration, and introduced under the epidermis by means of a lancet, it produces the following effects. During the first four-and-twenty hours the puncture becomes more or less inflamed ; from the second to the third day it is accompanied with slight tumefaction, and presents the appearance of a small papula surrounded with red areola ; from the third to the fourth day the disease assumes a vesicular form, the epidermis being raised by a fluid more or less opaque, presenting at its apex a small dark point ; from the fourth to the fifth day the contents of the vesicle become purulent, the apex of the pustule depressed, resembling very much the pustule of small-pox. At this period the areola, which had progressively increased, begins to diminish or altogether disappears, particularly if the disease does not increase : after the fifth day, however, the subjacent and surrounding tissues, which hithero had undergone little or no modification or were merely slightly œdematous, become indurated by the extravasation of a plastic lymph, which communicates to the touch the resistance and elasticity of cartilage. After the sixth day the contents of the pustule thicken, the pustule itself shrivels up, and is covered with crusts. These enlarge towards their base, and forming by successive strata, at length assume the form of a truncated cone with a depressed apex. If these crusts are detached, or if they fall off, we find under them an ulcer with the hard base of which we have spoken, extending through the whole thickness of the skin. The surface of this ulcer, of a deep red colour, is foul, covered with a thick adhesive pultaceous matter, almost like a false membrane, which cannot be removed by any attempt to clean the sore. The edges of the ulceration at this period appear as though it had been dug out from the surrounding parts by a sharp circular instrument. The immediate vicinity of the sore is surrounded by a red, dark, or livid margin, more elevated than the surrounding parts.

M. Ricord further establishes that chancre in its commencement is purely a local disease ; that constitutional or secondary affections can only take place after this antecedent ; that they do not occur in all cases, and only after the lapse of a certain period of time.

Whatever may be the varieties and complications which subsequently follow or accompany the inoculated chancre, the progress of the latter is in all instances

---

* " It would appear that these views were likewise entertained by Dr. Wallace, who divides chancre into two distinct stages or phases, the first one of ulceration, the second one of reparation ; he particularly insists upon the impropriety and danger of administering mercury during the first stage, that of ulceration."

such as we have described it. The pustular form of incipient chancre is only wanting when the parts to which the virus is applied are destitute of epidermis or epithelium, and it is only preceded by phlegmonoid inflammation when the matter has been introduced into the subcutaneous cellular tissue, or into the lymphatic system.

The ulcerations completely destroyed or arrested on the third, fourth, or fifth day from the application of poison are not liable to secondary inflammation. It is not before the fifth day that the induration of chancres commonly commences, and it is the indurated chancre that is most frequently followed by secondary symptoms; this induration seems to indicate that the affection has become in some measure already constitutional; as long as there is no induration we may suppose the disease to be merely local.

The varied appearance which primary venereal sores presents (says M. Ricord) has given rise to arguments against the identity of the venereal virus, and has led to the promulgation of the theory of a plurality of venereal poisons. Inoculation, however, sets this matter at rest, for whatever may be the actual character of the sore from which we take the pus, provided it be taken during the first stage of chancre, that of ulceration or indolence, we obtain by inoculation a regular pustule when the matter is introduced beneath the epidermis or epithelium; an ulcer when it is applied to a denuded surface; and an abscess when introduced into the cellular tissue, or into the lymphatic system.

The various characters of chancres or primary venereal sores, are due to circumstances which are foreign to the specific cause which produced them; these are principally the particular constitution of the patient, his mode of living, the influence of any antecedent or present disease with which he may happen to be affected, and not least the local treatment of the sore. It is from one or many of these circumstances that we see phagedenic ulcers in subjects who have contracted their disease from others affected with ulcers of the simplest character.

The first stage of chancre, i. e. of ulceration or indolence, is the only one during which the disease is susceptible of propagation by inoculation; the period of this stage is not limited, hence, M. Ricord has known primary venereal sores capable of propagation after having continued eighteen months." 34.

*Barbarous Operation for Phymosis.*—Mr. Parker gives, and apparently approvingly, a full account of M. Ricord's operation for phymosis. Here it is.

"Permanent phimosis, with excessive length of the prepuce, demands the operation of circumcision, unless, (says M. Ricord,) we remedy one deformity by the production of another, in performing the old operation of merely slitting up the prepuce with the knife. If there exist between the glans and prepuce recent and not very firm adhesions, these should be removed by dissection; when the adhesions are firm, it will be necessary only to circumcise as much of the prepuce as may leave free the meatus urinarius; vegetations should be removed with the redundant portion of the prepuce. The old operation, which consists in merely slitting up the prepuce, and removing the angles from the divided prepuce on either side, is liable to many objections, the chief of which is the deformity which the operation occasions; when this plan is deemed advisable, as it may be in persons who have naturally a short prepuce, M. Ricord removes a portion in the shape of the letter V, the apex which corresponds to the base of the glans, the base or open part being taken from the body of the prepuce.

In most cases M. Ricord prefers the operation of circumcision, which is to be performed in the following manner. The penis hanging in a relaxed state, a line is to be traced round it with ink, two lines in front of the base of the glans; this being done, the prepuce is to be drawn forwards, and fixed between the blades of a pair of dressing forceps, which are to be placed in front of the glans, and immediately behind the ink lines; the forceps are to be held perpendicularly and not transversely, and fixed in this position by an assistant. The operator then seizes the prepuce with his left hand and with a scalpel or bistoury, held in his right, cuts off that part of the prepuce, placed before the forceps, the latter serving as a guide for the direction of his incision. That portion of the mucous membrane, which has not been drawn forwards with the skin of the prepuce, is afterwards to be divided, with the view of preventing a secondary phimosis, or paraphimosis. The latter part of the operation is to be performed by dividing the mucous membrane with the scissors upon the dorsal aspect of the glans, and as far as its base; the two flaps thus made are to be dissected off on either side to the frenum, and removed with the latter by one stroke of the knife or scissors.

Some hemorrhage, from the dorsal artery of the penis, that of the frenum, or other small branches demands occasionally torsion of the arteries, or the use of the ligature. The penis should be covered with compresses soaked in cold water or the lead lotion, with the view of keeping down inflammation, and preventing erections. With the latter view also opiates with camphor should be administered." 157.

God help the penises that fall under M. Ricord's knife. He carves them as if he were cutting out a pattern of a penis, instead of a living, retiring, sensitive penis itself. One would think that penises were not made of flesh, so penal are his operations. This may do very well for a Parisian Hospital, but we can tell Mr. Parker that English gentlemen don't like their concerns so interfered with. What the hard-handed people of Birmingham may submit to, we can't say, but we know that in London such bloody-mindedness would not be put up with. If a man has a phymosis, he does not, in most instances have it cut for beauty's sake, but because the phymosis is an inconvenience. He wants as little of the knife as may be, and any surgeon who begins to slash so unmercifully as M. Ricord and Mr. Parker, will be *cut* himself, he may rely upon it.

We have operated, we will venture to say, on more phymoses than Mr. Parker, and we find the old plan answer well enough. The superabundant skin is, in most instances, greatly reduced; and after a year or two, it would hardly be known that any operation had ever been performed. Two or three times we have resorted to circumcision, but we have always found the patient complain, and that very bitterly, of the severity of the proceeding. Mr. Parker, we suspect, will find when he has much practice on private patients in this country, that a little *tenu* with the knife becomes requisite.

*Bubo.*—We need scarcely say that this may be simple, or "sympathetic," or it may be specific. Ricord, says Mr. Parker, has instituted the test of inoculation as a means of differential diagnosis between the two. The virulent bubo—that arising from the absorption of pus from a chancre, is a disease precisely similar to chancre, differing from it only in its seat, and in the anatomical organization of the parts in which it is seated. The true venereal bubo is the only one which gives a characteristic pustule by inoculation; and is the only certain means of enabling us to determine whether a bubo is venereal or sympathetic. In cases where bubo occurs as a primary symptom, this test becomes of the utmost importance, since by its results alone can we be led to frame a rational plan of treatment.

M. Ricord maintains that those only which furnish the characteristic pustule of chancre by inoculation are capable of being followed by secondary symptoms. Those from which no pustule can be obtained by inoculation are simply inflammatory, and must be treated on general principles.

With reference to the test of inoculation itself, some degree of difference of opinion exists, although M. Ricord states that the reason of this is, that the experiments have not been made in a proper manner.—On this point we consider this author's opinions worthy of attention. Whenever inflammation and suppuration of the cellular tissue, or lymphatic glands of the groin is owing to any other cause than the occurrence of chancre, the pus secreted furnishes no result from inoculation, at whatever periods and under whatever circumstances the test may be made. Neither does it follow, of necessity, that buboes succeeding to true chancres will furnish a specific pus; and consequently, by inoculation, a characteristic pustule. That this may occur it is necessary that the bubo shall not merely be owing to a simple sympathetic inflammation, but that actual absorption of the specific matter of the chancre shall have taken place. When absorption of the matter from a chancre on the genitals takes place, it is generally confined to the superficial glands of the groin; and most frequently the syphilitic poison is conveyed to one gland only, although many of the glands in the immediate vicinity of the latter, both superficial and deep seated, are inflamed, and suppurate at the same time, so that the matter taken from one gland shall be purely syphilitic, and give rise, by inoculation, to the characteristic pustule, whilst those in its immediate neighbourhood, and the cellular tissue, shall be affected by simple phlegmonoid inflammation, the pus from which shall, when tested by inoculation, give a negative result.

It may be very readily conceived, that the irritation produced by the passage of the syphilitic poison through a lymphatic vessel and ganglion may excite in the neighbouring organs an inflammation which is not specific, but merely phlegmonous, and this appears to be the true nature of the case. M. Ricord opened a bubo which had succeeded to a chancre, the pus from which produced no result by inoculation. In the centre of the abscess he discovered an enlarged lymphatic gland, presenting an evident fluctuation; this was punctured and tested by inoculation, the characteristic pustule of chancre was obtained.

It appears to us that the test of inoculation, even on M. Ricord's own shewing, (and we are far from taking all these facts upon trust,) is not worth much. It will scarce hold water after all. For *first*, we think that bubo should seldom suppurate at all—*secondly*, even though it be a bubo from a "chancre," it need not necessarily be specific—and *thirdly*, there are all the fallacies that may have waited on M. Ricord's experiments, and may wait on our own to be considered. Does M. Ricord mean to say that no secondary symptoms follow bubo from chancre, unless it will furnish the "test" of inoculation? How then does he account for those cases of secondary symptoms where there has been no bubo at all? If the virus can be absorbed without occasioning any inflammation of the gland, it can surely be absorbed without occasioning *specific* inflammation of the gland. The whole affair is a matter of moonshine. We should be sorry to pin our faith on M. Ricord. In a memoir he asserted that venereal ulcers deep in the vagina were quite common. We have examined a large number of women with the speculum, and we have *not* found such ulcers common; very far from it. We warn our readers to receive with great caution these positive assertions on the results of inoculation.

We turn to the treatment of bubo.

"In the first stage of bubo, when the inflammatory symptoms are not marked, M. Ricord recommends rest and compression: this author has remarked that, in patients wearing trusses buboes are seldom if ever developed on the side where the pressure of the truss is acting, but on the opposite one; hence in the first stage of bubo, that of mere enlargement, without any acute inflammatory action or pain, a well regulated pressure, by means of an appropriate bandage or apparatus, is frequently successful in dispersing the tumour. This plan of treatment is above all useful in the bubou d'emblée. It must be associated with an antiphlogistic regimen, rest, and gentle aperients.

The same plan of treatment may be followed in the treatment of the true syphilitic bubo, unattended by much pain or inflammation. In this stage, unless specially contra-indicated, mercury may be employed to assist the resolution of the tumour. The primary syphilitic bubo, may (says Dr. Wallace), in its first stage, be resolved in ninety-nine cases out of a hundred by mercury; if this medicine be used after the plan recommended for primary syphilis, and if its operation be assisted by rest, laxatives, abstinence, and cooling lotions. It is well, in reference to this opinion, to remark, that a vast number of those buboes which succeed to true chancres are sympathetic, that is, when they suppurate, they do not furnish or secrete a specific pus. Hence it must be evident that the general employment of mercury is, to say the least, unnecessary, except so far as it may be used with a view of controlling inflammatory action. Cullerier thinks that at this period, uncertain as we must be as to the true character of the bubo, that it should be treated as a pure and simple inflammation. When accompanied by chancre it is of vast importance to our success in the resolution of the bubo, to allay all pain or irritation which may exist in the sores themselves, and for this purpose the aqueous solution of opium before recommended will be found of great service.

When the commencement of bubo is accompanied by much pain, tenderness on pressure, or heat of parts, the local abstraction of blood may be necessary, although I have not a high opinion of this measure in the resolution of bubo generally. It may even be necessary to bleed from the arm if the patient be plethoric, and the local disease associated with general excitement, or much symptomatic fever. In local bleedings thus employed, it will be found advantageous to apply a small number of leeches, from four to eight, or more, and wait till the oozing of blood begins to cease, then to apply another relay of leeches so as to keep up a constant draining of blood from the part for twelve or more hours. This form of bleeding, termed 'permanent,' is found to reduce the inflammation more certainly and speedily than the application of a large number of leeches at once. Two, three, or more relays of leeches may be thus employed, proportionate to the strength of the patient and the intensity of the local disease." 105.

We have tried compression, but we must confess that it disappointed us. First of all, it is not very easily regulated satisfactorily, being either too great or too little. Secondly, it has seemed to us in many instances to aggravate inflammation, rather than to disperse it. Compression has, in our hands, proved most serviceable in cases of indolent bubo, not of incipient bubo.

And of mercury we must say that it should be used more sparingly than Mr. Wallace advised. There are many cases, where the bubo displays little tendency to inflammation, in which mercury acts very well. But it will often happen that the bubo grows worse under its use. Perhaps the best rule is not to give it, if there is much inflammation; or to discontinue it if the bubo increases during its employment.

We agree with Mr. Parker that great leeching is seldom of great service. At the same time we would not, as some do, proscribe it altogether.

There cannot be a doubt of the occasional utility of blisters. Even when suppuration seems certain and extensive we have seen them cure bubo. But we have often seen them fail, and, though a good, we cannot but confess that they are an uncertain remedy; for they fail in cases, to all appearance, similar to those in which they answer. We *think* we have made out that they are not applicable to bubo much inflamed—nor to incipient bubo—nor to persons of a very irritable habit. But we would not speak very confidently upon any point, and, possibly, the best practical rule is this, to *try* a blister when the bubo is advancing in spite of other remedies. It may do great good, and it cannot do much harm. Even if the bubo suppurates it facilitates the pointing, and limits, we fancy, the extent of the abscess.

" In the treatment of indolent bubo, in the commencement, M. Ricord has recourse to the discutient plasters with compression in the day time, and friction with the iodide of potassium, or mercurial ointment, in the evening, covering the part during the night with a poultice. If this has not a marked effect upon the enlargement in a few days, blisters with the bichloride of mercury on Malapert's plan are employed. Frictions with ointments composed of the proto-ioduret of mercury,* or compresses soaked in a dilute tincture of iodine,† are also very useful in the resolution of the chronic or indolent bubo." 110.

We have found the tincture of iodine serviceable in chronic bubo. It occasions a series of desquamations of the cuticle, which appears to promote the resolution of the gland. Sarsaparilla and the liquor potassæ, the latter in full doses, are of great utility.

" The disease may terminate in two ways: the enlarged glands may pass on slowly to suppuration, or assume a form of induration of a scirrhous or scrofulous character. In the latter form, friction with croton oil, the tincture of iodine, or the emp. belladonnæ with the tartar emetic, may be used as local applications. Small issues may also be formed over the induration, by means of the caustic potash, or if the disease assume a purely scirrhous character, extirpation may become necessary."

We cannot say that we have seen buboes grow scirrhous. A pregnant instance this of the loose way in which " scirrhus" is viewed by continental surgeons. On all that relates to the malignant alterations, they are many years behind us. We need scarcely say that we do not patronise the extirpation of such glands, and we would caution Mr. Parker's readers and Mr. Parker himself against indulgence in it.

An early opening and a free opening is the best for the *bubonic* abcess. If the skin is very bad, the potassa fusa may be used, but we believe that with judicious management, the skin generally recovers itself after a proper opening with the knife.

---

* ℞. Hydrargyri proto-ioduret, ℈ j.

Adipis, ℥ j. M. ft. Unguent.

† ℞. Tinct. iodinii, ℥ j.

Aquæ distillat, ℥ ij. M. ft. Lotio.

